# Analyzing Evidence on Interventions to Strengthen the Clinical Support for Midwifery Students in Clinical Placements: Protocol for a Systematic Scoping Review

**DOI:** 10.2196/29707

**Published:** 2021-09-21

**Authors:** Hafaza Amod, Sipho Wellington Mkhize, Claudine Muraraneza

**Affiliations:** 1 Discipline of Nursing School of Nursing and Public Health University of KwaZulu-Natal Durban South Africa

**Keywords:** midwifery students, registered midwives, clinical support interventions, midwives, midwifery, students, mentorship, clinical supervision, collaboration, clinician attitudes

## Abstract

**Background:**

The benefits of clinical support are evident in various mentorship, preceptorship, or clinical supervision models. Poor collaboration between lecturers and clinical staff, lack of confidence about student support, large student intakes coupled with core demands create negative attitudes toward student supervision, and this poses a huge challenge to midwifery students who are expected to become competent in the process.

**Objective:**

This study aims to identify and analyze interventions, strategies, and/or mechanisms in order to strengthen the clinical support for midwifery students in clinical practice areas from a global perspective.

**Methods:**

This review will follow the Arksey and O’Malley framework (2005). The search strategy will include primary studies searched for in electronic databases such as EBSCOhost (CINAHL, MEDLINE, and Health Source: Nursing/Academic edition), PubMed, Google, and Google Scholar. Keywords such as “midwifery students,” “midwifery education,” and “clinical support” will be used to search for related articles. The search will include articles from the cited by search, as well as citations from the reference list of included articles. All title-screened articles will be exported to an EndNote library, and duplicate studies will be removed. Two independent reviewers will concurrently carry out the abstract and full-text article screening according to the eligibility criteria. Extracted data will highlight the aims, geographical setting, and level of training; intervention outcomes; and the most relevant and most significant findings. This review will also include a mixed methods quality appraisal check. A narrative summary of data extracted will be analyzed using content analysis.

**Results:**

Interventions to strengthen the clinical support for midwifery students in practice will be extracted from this review, and data will be analyzed and extracted to develop a comprehensive guide or framework for clinical mentorship. As of August 2021, the electronic search, the data extraction, and the analysis have been completed. The results paper is expected to be published within the next 6 months.

**Conclusions:**

It is expected that this review will contribute to midwifery education by identifying quality evidence on clinical support interventions available to midwifery students globally, as well as best practice methods, procedures, or interventions that can be used to develop a midwifery mentorship training program.

**International Registered Report Identifier (IRRID):**

DERR1-10.2196/29707

## Introduction

The clinical support for midwifery students is critical to the quality of graduates produced at higher education institutions. A significant concern for lecturers and registered midwives is to produce graduates who are safe and competent practitioners [1-3]. Midwifery students spend 50% of module time in clinical placements for work-integrated learning. Therefore, a registered midwife who supports students in clinical placements has an extremely important role to create and maintain a positive working experience, increasing students’ enthusiasm and ensuring their retention in the profession [4-6].

Midwifery students value the clinical support they receive during their transition from a student to a confident midwife practitioner. The benefits of clinical support are evident in various mentorship, preceptorship, or clinical supervision models, and it is supported widely in the literature [7-9]. However, literature on the perceptions of mentors or preceptors concurs that clinical staff feel unprepared in their roles to support students in clinical placements [10-15]. Furthermore, time constraints and the core function of registered midwives, which is to deliver patient care, hampers opportunities to support students during clinical placement for learning [15].

Findings from other studies also showed positive outcomes in the student-mentor relationship, even more so when mentoring is undertaken in a planned method [3,6]. In addition, providing support and training to registered midwives to take on the role of a clinical mentor or preceptor is highly recommended in many developed countries such as New Zealand, Scotland, and the United Kingdom [15-18]. Very few studies conducted in African countries relate to the clinical support for midwifery students [2,19]. One study called the MOMENTUM project was conducted in Uganda and supported by the Royal College of Midwives (United Kingdom). The project aimed to address the poor quality of mentorship for midwifery students by developing a context-specific model for mentorship in Uganda [19].

In South Africa, registered midwives working in clinical placements assume the role of clinical mentors. These clinical mentors do not receive any formal support or training and, therefore, experience conflicts in their roles and expectations. Poor collaboration between lecturers and clinical staff, negative feelings, lack of confidence about student support, and large student intakes create negative attitudes toward clinical supervision [2,20]. Currently, in South Africa, there are no known support structures for registered midwives who support students in clinical practice. Hence, the quality of midwifery mentorship is questionable, and the need to train and support registered midwives to mentor students in maternity care units has become necessary.

Identifying and analyzing the interventions to support mentorship training on a global capacity has not been previously conducted in South Africa. There are also no scoping reviews on clinical support structures or interventions to strengthen midwifery clinical support. The results of this systematic scoping review will identify interventions to strengthen the clinical support for midwifery students; subsequently, through data analysis, these results could help in developing a comprehensive mentorship training guide for midwifery clinical practice.

## Methods

### Study Design

This systematic scoping review will focus on retrieving and reviewing studies on clinical support interventions available to midwifery students globally. The review will follow the Arksey and O'Malley (2005) framework [21] using the following steps: (1) identifying the research question; (2) identifying the relevant studies; (3) study selection; (4) charting the data; (5) collating, summarizing, and reporting the results; and (6) consultation (optional).

### Objectives

The objective for this systematic scoping review is to identify and analyze best practice guidelines, interventions, strategies, and/or mechanisms in order to support midwifery students in clinical practice areas on a global perspective.

### Identifying the Research Question

What evidence is available on interventions to strengthen the current clinical support for midwifery students globally?

### Eligibility of the Research Question

The review will use the population, concept, context (PCC) framework, as described by Levac et al [22,23], to determine the research question’s eligibility criteria. Table 1 shows the eligibility criteria and the elements to be used in the review. 

**Table 1:** The population, concept, context framework.

Eligibility criteria	Elements of the study
Population	Studies that include training of midwifery undergraduate and/or postgraduate students. Studies that include the perspectives of mentors and mentees.
Concept	To strengthen clinical support for midwifery students. Clinical support terms such as “clinical supervision,” “mentorship,” and “preceptorship” are used interchangeably in nursing and midwifery practice.
Context	Midwifery education and training, globally.

### Identifying Relevant Studies

This scoping review will select preliminary studies using qualitative, quantitative, and mixed methods related to clinical support for midwifery students. Electronic platforms such as EBSCOhost (CINAHL, MEDLINE, Health Source: Nursing/Academic Edition), PubMed, Science Direct, Google, and Google Scholar will be searched to find articles published in peer-reviewed journals and the grey literature. The search strategy involves using search terms such as “midwifery students,” “clinical supervision OR mentorship OR preceptorship,” and “midwifery education.” The search will be limited to English-language articles and confined within the last 10 years (2010-2020) to identify support interventions and strategies that are up to date and current. 

The review will include a manual search of the main published articles and citations from the “related literature” list. Eligibility criteria to ensure specific information relating to the research question will be used in the studies. It will include Boolean terms (“midwifery AND clinical support,” OR “mentorship,” OR “clinical supervision,” OR “preceptorship”), medical subject headings (MESH) terms (“midwifery students AND clinical support interventions,” “mentorship AND midwifery students,” and “midwifery practice and clinical supervision models”). If full-text articles are unobtainable, the researchers will consult with the librarian for assistance. All researchers will maintain an electronic search record of all literature searched.

### Study Selection

The researcher will design a form for abstract and full-text screening by using Google Forms. The search strategy will follow a 3-stage system of title screening, abstract screening, and full-text screening, as determined by the inclusion criteria mentioned below. All selected articles from the screening process will be saved in an EndNote software folder.

#### Inclusion Criteria

The following studies will be included: (1) studies that present evidence on midwifery students; (2) studies that present evidence on clinical support such as mentorship, preceptorship, and clinical supervision; (3) studies that present evidence on midwifery education; (4) studies conducted between 2010 and 2020; (5) studies that include a support intervention or strategy; and (6) peer-reviewed articles and studies from the grey literature, which may include governmental policies and guidelines.

#### Exclusion Criteria

The following studies will be excluded from the analysis: (1) studies that do not include midwifery students and (2) studies that do not include an intervention or strategy.

#### The Screening Process

The primary investigator will conduct a thorough title-screening process using relevant databases. All articles selected will be exported to an EndNote library. Duplicated articles will be extracted from the reference list. The primary investigator and an independent collaborator will screen all saved abstracts using a standardized Google Forms as a tool. Both the primary investigator and the independent collaborator will apply the inclusion criteria developed for the search. The eligible articles selected from the abstract-screening stage will then undergo a full-text article screening process using another standardized Google Forms. Both the primary investigator and the research collaborator will work independently. Both screeners will also compile a screening report for both the abstract and full-text screening. A third reviewer (the research supervisor) will resolve any discrepancies that may emerge. 

### Charting the Data

In this stage, the researcher will design a data charting tool using Google Forms. Textbox 1 shows the variables used in the data charting tool. The data charting tool will highlight the study’s aims, intervention outcomes, the most relevant findings, and the most significant findings, and author comments.

All researchers will collectively conduct a content analysis to extract relevant outcomes. All emerging themes and variables will be used to answer the research question. The data charting tool will be updated continually.

**Variables used in the data charting stage.**

**Variables used in the data charting form:**

- Author and date
- Full journal reference
- Study aims or research question
- Geographical setting
- Level of training
- Intervention outcomes (methods, procedures, evaluation, removal and monitoring, preferences, and acceptability)
- Most relevant findings
- Most significant findings
- Comments

### Quality Appraisal

This study will include a quality check as recommended by Levac et al [23]. A mixed methods quality appraisal tool designed by Pluye et al [24] will be used to assess the methodological quality of studies retrieved. According to the mixed methods quality appraisal tool, there are 4 different criteria used in both qualitative and quantitative study designs and 3 criteria used in the mixed methods section. A scoring metrics system will present all outcomes according to the number of criteria met. Table 2 shows an example summary of the scoring metric, presented according to the study design, the number of criteria met, and the percentage score; the corresponding descriptors will be recorded alongside.

A score of 75% and higher indicates a high-quality outcome and will be included in the study. A score of 25% and below indicates a low-quality outcome and will not be included in the study.

**Table 2:** Scoring metrics summary (example).

Study design and number of criteria met		Score (%)	Descriptors
**Qualitative and quantitative studies**			
	1	25	*
	2	50	**
	3	75	***
	4	100	****
**Mixed method studies**			
	0	25	*
	1	50	**
	2	75	***
	3	100	****

### Collating, Summarizing, and Reporting the Results

**Figure 1:**
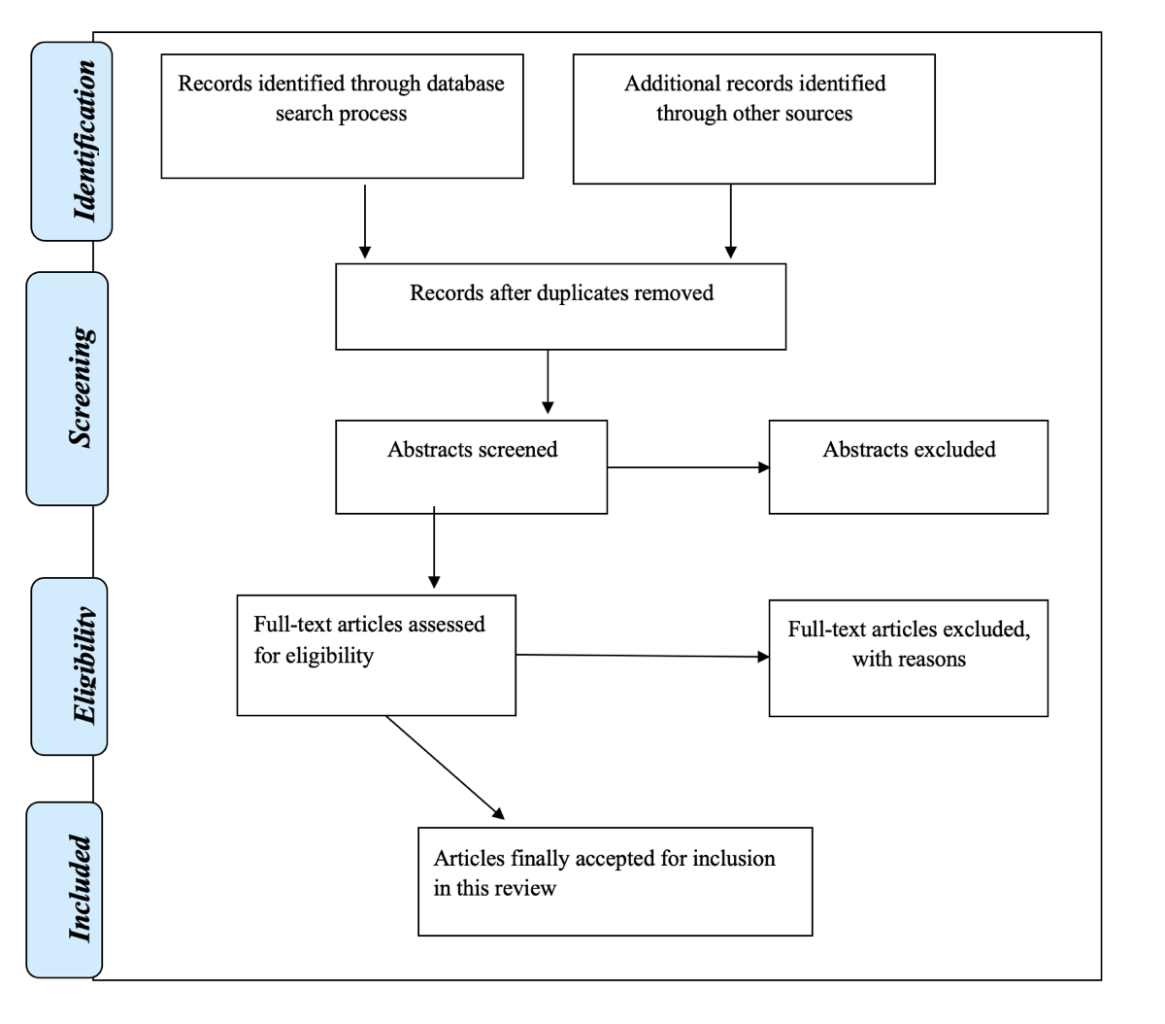
PRISMA (Preferred Reporting Items for Systematic reviews and Meta-analyses) flow diagram presenting screening results.

A narrative summary of data extracted will be analyzed using content analysis. Only the most relevant and most significant data in line with the research question will be included in the study. The results of the systematic scoping review will be mapped in a 2009 PRISMA (Preferred Reporting Items for Systematic reviews and Meta-analyses) flow diagram, as shown in Figure 1. Once the protocol is accepted, the systematic scoping review findings will be published in an accredited journal in an electronic format. Results will also be presented at midwifery and nursing education conferences nationally and/or internationally.

### Ethics Approval and Consent to Participate

The study was approved by the affiliated university’s ethics committee for Human Social Science (Ethics approval no. HSS/1509/018M).

### Availability of Data and Materials

All data generated and analyzed from this study will be included in the published systematic review article and will be available on request.

## Results

Interventions to strengthen the clinical support for midwifery students in practice will be extracted from this review, and data will be carefully analyzed to develop a comprehensive guide or framework for clinical mentorship. As of August 2021, the electronic search, the data extraction, and the analysis have been completed. The results paper is expected to be published within the next 6 months.

## Discussion

The quality of clinical support for midwifery students in placement learning is well debated as some clinical staff feel unprepared to instruct new students [12,13]. Mentors play a vital role in shaping students as qualified midwives, and the mentor-student relationship affects confidence in practice [25,26]. Thus, the poor support received during clinical practice may lead to inadequately prepared graduates who contribute to the high maternal mortality rates, especially in African countries such as Botswana, Lesotho, Swaziland, Zimbabwe, Malawi, Namibia, Mozambique, Angola, and South Africa.

According to the 2008 Nursing and Midwifery Council (NMC) requirements, trained mentors undertake assessments and provide feedback on preregistration midwifery students' proficiencies. This expectation can be especially useful in the South African context, as students have to fulfill long hours in clinical placements to achieve clinical requirements and hours. However, contrary findings were found in other studies using the same, abovementioned requirements. Studies found that mentors had difficulties assessing, supervising, supporting, and guiding students in practice [11,27-29].

The fundamental aim of midwifery education is to develop a safe and competent practitioner who will resume full responsibility and accountability for practice [30]. Ensuring that midwifery students are equipped with the necessary skills to provide high standards of care remains a challenge for lecturers and clinical mentors. Therefore, reviewing and analyzing best practice interventions, strategies, or models that strengthen clinical support for midwifery students is urgently needed.

This systematic scoping review aims to review and analyze the current clinical support systems available to midwifery students globally and identify a suitable intervention to strengthen clinical support for midwifery students in South Africa.

## Abbreviations

NMC: Nursing and Midwifery Council
PCC: population, concept, context
PRISMA: Preferred Reporting Items for Systematic reviews and Meta-analyses
